# The impact of subclinical hypothyroidism on core reproductive hormones and ovarian endocrine status in patients with polycystic ovary syndrome: a systematic review and meta-analysis

**DOI:** 10.3389/fendo.2026.1893428

**Published:** 2026-09-11

**Authors:** Yunhui Liu, Tingting Deng, Ping Zhao

**Affiliations:** Department of Endocrinology, Liuzhou Workers’ Hospital, The Fourth Affiliated Hospital of Guangxi Medical University, Guangxi, China

**Keywords:** meta-analysis, polycystic ovary syndrome, reproductive hormones, subclinical hypothyroidism, systematic review

## Abstract

**Objective:**

This systematic review and meta-analysis aimed to evaluate the effects of subclinical hypothyroidism (SCH) on serum levels of reproductive hormones, including follicle-stimulating hormone (FSH), luteinizing hormone (LH), estradiol, prolactin, and total testosterone, in women with polycystic ovary syndrome (PCOS) and to provide evidence for clinical practice.

**Methods:**

We searched PubMed, Web of Science, Embase, and Cochrane Library from inception to 25 April 2026 for relevant observational studies. Study quality was assessed using the Newcastle–Ottawa Scale (NOS). Statistical analyses were performed using R software, with the standardized mean difference (SMD) and 95% confidence interval (CI) used as effect measures. Heterogeneity was evaluated using the I² statistic, followed by subgroup analysis, sensitivity analysis, and publication bias tests.

**Results:**

Thirteen studies involving 3,114 participants (579 PCOS patients with SCH and 2,535 euthyroid PCOS controls) were included. Pooled analyses showed no statistically significant differences in serum FSH, LH, estradiol, or total testosterone levels between groups. For prolactin, a significant elevation emerged after *post-hoc* exclusion of an outlying study contributing to extreme heterogeneity; this finding is exploratory. No significant publication bias was detected for FSH and LH; however, tests were underpowered for the other three outcomes, and Begg’s test suggested potential small-study effects for prolactin. Certainty according to the Grading of Recommendations Assessment, Development and Evaluation (GRADE) framework was low for FSH and very low for LH, estradiol, prolactin, and total testosterone.

**Conclusion:**

Current evidence shows no significant differences in FSH, LH, estradiol, or total testosterone levels between SCH and euthyroid PCOS controls, while the evidence for prolactin remains uncertain. High heterogeneity and the observational study designs preclude definitive conclusions or causal inference.

**Systematic Review Registration**

https://www.crd.york.ac.uk/PROSPERO/view/CRD420261382326, identifier CRD420261382326.

## 1 Introduction

Polycystic ovary syndrome (PCOS) is the most common endocrine and metabolic disorder in women of reproductive age. It is characterized by ovulatory dysfunction, hyperandrogenism, and polycystic ovarian morphology, and it increases the risks of infertility, menstrual irregularity, and metabolic syndrome (1, 2). Subclinical hypothyroidism (SCH) is a common thyroid disorder defined by elevated serum thyroid-stimulating hormone (TSH) with normal free thyroxine (FT4) levels (3). In recent years, the prevalence of comorbid PCOS and SCH has increased significantly in women of reproductive age due to changes in lifestyle and dietary patterns (4, 5). This comorbidity complicates clinical management and has sparked extensive debate over whether SCH is associated with more severe reproductive endocrine disturbances in patients with PCOS.

To date, the quantitative association between SCH and core reproductive hormone disturbances in PCOS remains highly disputed across clinical observational studies and prior published meta-analyses (6, 7). The landmark 2017 meta-analysis by Pergialiotis et al. (8), which is widely cited in clinical discussions of thyroid screening for PCOS patients, synthesized only literature published before 2016, evaluated only follicle-stimulating hormone (FSH), luteinizing hormone (LH), and sex hormone-binding globulin (SHBG) without a comprehensive pooled analysis of estradiol, prolactin, and total testosterone, and lacked stratified analysis based on clinically variable TSH diagnostic cutoffs or thyroid autoimmunity status. A 2018 meta-analysis by Ding et al. (9) focused exclusively on estimating the pooled prevalence of SCH among PCOS populations and contained no quantitative synthesis of reproductive hormone profiles; therefore, it could not address conflicting endocrine outcomes across primary studies. Another large-scale meta-analysis published by Xing et al. in 2021 (10) included 27 studies but relied heavily on Chinese gray literature. The study neither applied the Grading of Recommendations Assessment, Development and Evaluation (GRADE) framework for certainty rating nor conducted rigorous leave-one-out analysis or risk-of-bias-stratified sensitivity analysis to explore between-study heterogeneity. Other narrative reviews published in 2022 provided only qualitative summaries without pooled quantitative effect sizes, thus offering limited actionable guidance for clinical endocrine evaluation (11).

Two recent reviews identified major gaps in the existing evidence. A 2022 systematic review pooled global cross-sectional data and showed conflicting correlations between SCH and reproductive hormones across ethnic cohorts, with no updated quantitative synthesis to resolve these discrepancies (7). A 2023 review outlined thyroid–ovarian axis crosstalk and identified thyroid autoantibody positivity and inconsistent TSH cutoffs as two key confounders driving high between-study heterogeneity; however, no meta-analysis had performed stratified quantitative analyses for these two factors at the time of publication (12). Multiple new observational cohorts from various countries have been published between 2021 and 2026, all falling outside the search windows of the three prior meta-analyses, creating a substantial time lag in the current evidence base. Most original studies rely on single-center, small-sample designs without unified hormone testing protocols or standardized confounder adjustment. These inconsistencies fragment the available data and hinder clinicians from establishing standardized endocrine monitoring plans for PCOS patients with SCH.

This systematic review and meta-analysis was therefore performed to integrate updated observational evidence and examine the associations between SCH and serum levels of FSH, LH, estradiol, prolactin, and total testosterone in women with PCOS. We aim to describe the current evidence on differences in hormone levels and identify methodological gaps requiring further investigation.

## 2 Materials and methods

### 2.1 Study registration

This systematic review and meta-analysis was designed and performed in accordance with the Cochrane Handbook for Systematic Reviews of Interventions. We followed the Preferred Reporting Items for Systematic Reviews and Meta-Analyses (PRISMA) statement (13). The review protocol was formally registered on 1 May 2026 in the International Prospective Register of Systematic Reviews (PROSPERO) (ID: CRD420261382326), publicly available at https://www.crd.york.ac.uk/prospero/display_record.php?ID=CRD420261382326.

Database searching was completed on 25 April 2026, with only citation export performed at that stage. All subsequent screening, data extraction, subgroup analyses, and sensitivity analyses were performed according to the registered protocol after registration, with no *post-hoc* adjustments to outcomes or analytical plans after observing the pooled results.

### 2.2 Inclusion and exclusion criteria

The population, exposure, comparator, and outcomes (PECO) framework, which is commonly used for observational systematic reviews, was used to define the research question and eligibility criteria (**Table 1**). Eligible study designs included cross-sectional, case-control, and retrospective cohort studies. Only original studies reporting means and standard deviations (SDs) (or data that could be converted to means and SDs) for the above outcomes were included. Reviews, letters, conference abstracts, animal studies, and studies without extractable outcome data were excluded.

**Table 1:** PECO framework for study eligibility screening.

Dimension	Detailed definition
Population (P)	Women of reproductive age with a confirmed diagnosis of PCOS based on standard clinical diagnostic criteria
Exposure (E)	Concomitant SCH, defined as elevated serum TSH above the laboratory reference cutoff with normal FT4 levels.
Comparator (C)	Euthyroid PCOS controls (with normal TSH and normal FT4 and no SCH).
Outcomes (O)	The primary outcome was serum follicle-stimulating hormone (FSH) level, a marker associated with ovarian reserve. Secondary outcomes included a complete reproductive hormone profile: estradiol, LH, total testosterone, and prolactin.

The population, intervention, comparison, outcomes, and study design (PICOS) framework was developed for interventional trials and contains an intervention component unsuitable for this observational meta-analysis. PECO was applied herein to align with exposure-comparison study design, and all core eligibility dimensions of PICOS are integrated into the four PECO categories above.

### 2.3 Search strategy

Electronic searches were conducted in PubMed, Web of Science Core Collection, Embase, and Cochrane Library. All retrievals were completed on 25 April 2026 via each database’s official web interface and included all records from database inception to the cutoff date. Only peer-reviewed English-language journal articles were included.

We restricted our analysis to English-language publications for three reasons: high-quality relevant observational studies are predominantly published in English; the use of a single language avoids translation bias and inconsistent data extraction; and non-English regional papers are mostly small, single-center studies with variable methodology that may introduce additional heterogeneity. Nevertheless, this rule may create language bias by omitting eligible non-English regional publications.

Searches of trial registries and gray literature, including conference abstracts, dissertations, and preprints, and citation tracking were not performed. Unpublished or non-peer-reviewed materials usually lack complete outcome data and detailed methods, so we excluded these sources to improve the reliability of the pooled data.

The search syntax integrated Medical Subject Headings (MeSH)/Emtree controlled terms and free-text keywords connected by Boolean operators. Full replicable search strings for every database are listed in **Supplementary Table 1**.

### 2.4 Literature screening and data extraction

Literature screening and data extraction were independently performed by Yunhui Liu and Ping Zhao. All disagreements in screening judgments were first resolved through discussion; any remaining disagreements were adjudicated by the third reviewer, Tingting Deng. EndNote X9 was used to organize all retrieved citations and remove duplicates. The two reviewers separately screened titles, abstracts, and full texts offline according to predefined PECO eligibility criteria and then cross-checked their screening results. All extracted quantitative data were recorded in a standardized Excel form.

Extracted items included:

1. Basic study information: first author, publication year, country of recruitment, and study design type;
2. Participant baseline characteristics: total study sample size, number of PCOS patients with SCH, age, body mass index (BMI), use of the Rotterdam PCOS diagnostic criteria, and specific TSH cutoff thresholds used to define SCH;
3. Thyroid autoimmunity data: whether thyroid peroxidase antibody (TPOAb) and thyroglobulin antibody (TgAb) testing was performed, and whether reproductive hormone outcomes were separately reported for antibody-positive vs. antibody-negative SCH subgroups;
4. Measured reproductive hormone indicators: FSH, LH, estradiol, prolactin, total testosterone;
5. Raw quantitative outcome data: sample size, mean value, and standard deviation of each hormone for PCOS patients with SCH and euthyroid PCOS subgroups;
6. Confounder adjustment records: matching or multivariable adjustment status for key covariates including age, BMI, insulin resistance, PCOS phenotype, thyroid autoimmunity, medication history, infertility status, menstrual phase of blood collection, and standardized hormone testing protocols.

For studies that did not report standard deviation (SD) directly, values were estimated using methods recommended by the Cochrane Handbook from p-values, t-values, 95% confidence intervals (CIs), standard errors, or CI limits. If studies reported medians and interquartile ranges, data were converted to means and SDs using the methods of Luo et al. (14) and Wan et al. (15).

### 2.5 Risk of bias assessment

Different quality assessment instruments were applied according to study design: cross-sectional studies were evaluated using the 9-item Agency for Healthcare Research and Quality (AHRQ) checklist, whereas case–control and cohort studies were appraised with the Newcastle–Ottawa Scale (NOS) (16, 17). The raw total scores from the AHRQ checklist and NOS served only as supplementary information rather than as the primary metric for presenting risk-of-bias results. To improve the interpretability of the clinical findings, we further conducted independent evaluations of all eligible studies across eight key clinical domains: ① Participant selection; ② Definition and measurement of subclinical hypothyroidism (SCH);③ Diagnostic criteria for polycystic ovary syndrome (PCOS); ④ Comparability between the SCH group and the euthyroid PCOS control group; ⑤ Adjustment or matching for confounding factors; ⑥ Hormone assays; ⑦ Timing of blood sampling for hormone measurement; ⑧ Completeness of outcome data. Each domain was categorized as having a low, moderate, or high risk of bias.

### 2.6 Statistical analysis

All statistical analyses were performed in R 4.3.1 with the meta, metafor, and dmetar packages. For continuous outcomes, the standardized mean difference (SMD) was adopted as the primary effect size alongside the mean difference (MD), with 95% CIs, to unify units across studies. A random-effects model with DerSimonian–Laird τ² estimation was prespecified for all analyses because of the inherent clinical heterogeneity of observational data; fixed-effects models served as sensitivity checks. Heterogeneity was assessed using Cochran’s Q and I² (I² > 50% = substantial). We conducted Hartung–Knapp-adjusted random-effects sensitivity analyses and calculated 95% prediction intervals (PIs) for all outcomes to estimate expected effect ranges in future studies, with all PIs reported in the Results. Three predefined SCH subgroups were planned: a low TSH cutoff (~2.5 mIU/L), an adult standard TSH cutoff (4.0–5.0 mIU/L), and antibody-positive SCH. Additional leave-one-out and risk-of-bias-stratified sensitivity analyses were performed to explore sources of heterogeneity. Egger’s and Begg’s tests were used to evaluate publication bias. Certainty was evaluated using a Grading of Recommendations Assessment, Development and Evaluation (GRADE)-like framework adapted to observational evidence.

## 3 Results

### 3.1 Literature search results

**Figure 1:**
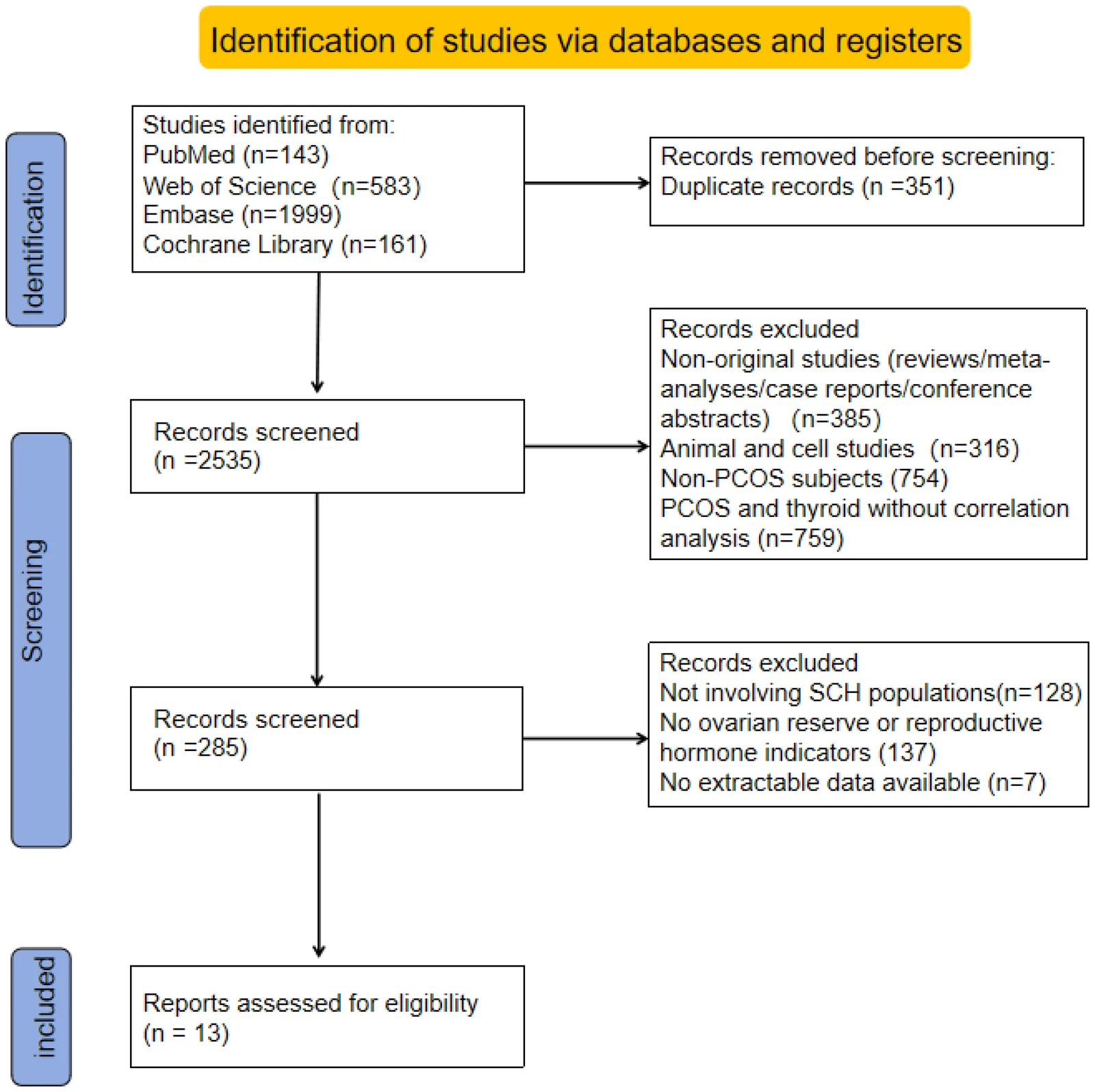
PRISMA flow diagram of the study selection process.

A total of 2,886 records were initially identified. After excluding 351 duplicates, 2535 records underwent title and abstract screening. Most ineligible articles were excluded because of inappropriate study types, irrelevant research subjects, and unrelated research themes, leaving 285 articles for full-text assessment. Further full-text screening excluded studies without SCH groups or valid reproductive hormone indicators. Seven studies with unextractable data were also excluded. Finally, 13 eligible studies were included in the systematic review and meta-analysis. The detailed screening process is shown in **Figure 1**. Full details of representative excluded studies and specific reasons for exclusion are provided in **Supplementary Table 2**.

### 3.2 Basic characteristics of the included literature

A total of 13 cross-sectional, case-control, and retrospective cohort studies (18–30) from nine countries involving 3,114 women with PCOS (579 with SCH and 2,535 euthyroid PCOS controls) were included; all studies used the Rotterdam PCOS criteria, whereas SCH was diagnosed using variable TSH cutoffs ranging from 2.5 to 5.0 mIU/L with normal thyroid hormone levels. The reproductive hormones measured were FSH, LH, estradiol, prolactin, and total testosterone, and most studies matched participants only by age and BMI without multivariable adjustment for key confounders, including insulin resistance and PCOS phenotype. Two studies (18, 27) tested TPOAb/TgAb thyroid autoantibodies, and merely Gawron et al. (27) separately reported reproductive hormone results for antibody-positive autoimmune SCH and isolated non-autoimmune SCH subgroups; the other 11 studies did not detect thyroid autoantibodies or provide antibody-stratified hormonal data, so quantitative subgroup meta-analysis based on thyroid autoimmunity status could not be carried out due to insufficient available stratified evidence (**Table 2**).

**Table 2:** Basic characteristics of the literature.

Author, year	Country	Study design	Sample size (total/SCH)	Age (years)	BMI (kg/m²)	PCOS criteria	SCH definition	TPOAb/TgAb	Outcome data	Confounder adjustment and matching
Ach 2025 (18)	Tunisia	Cross-sectional	161/24	Median 22 (IQR 20–26)	Median 29 (IQR 24.24–34)	Rotterdam	TSH > 4.5 mIU/L and normal FT4	Detection of TPOAb	FSH, LH, estradiol, prolactin	Age(M+A), BMI(M+A), IR(NR), PCOS phenotype(NR), TAI(NR), Medication(NR), Infertility(NR), Cycle(M), Assay(M)
Fatima 2020 (19)	Pakistan	Cross-sectional	90/31	24.6 ± 4.67 (SCH)	32.5 ± 3.75 (SCH)	Rotterdam	TSH > 2.5 mIU/L and normal FT4	Not performed	FSH, LH, total testosterone	Age(NR), BMI(NR), IR(NR), PCOS phenotype(NR), TAI(NR), Medication(NR), Infertility(NR), Cycle(NR), Assay(M)
				23.4 ± 4.54 (Control)	25.7 ± 3.27 (Control)					
Trakakis 2017 (20)	Greece	Case-control	280/21	Median 25 (15–37) (SCH)	Median 25(19–50)(SCH)	Rotterdam	TSH > 4 mIU/L and normal T3/T4	Not performed	FSH, LH, estradiol, total testosterone	Age(M), BMI(M), IR(NR), PCOS phenotype(NR), TAI(NR), Medication(NR), Infertility(NR), Cycle(NR), Assay(M)
				Median 24 (12–44) (Control)	Median 23(16–45)(Control)					
Hua 2025 (21)	China	Case-control	124/31	28.17 ± 3.45 (SCH)	23.56 ± 3.67 (SCH)	Rotterdam	TSH > 4.0 mIU/L and normal FT4	Not performed	FSH, LH, estradiol, prolactin	Age(M), BMI(M), IR(NR), PCOS phenotype(NR), TAI(NR), Medication(NR), Infertility(NR), Cycle(M), Assay(M)
				28.45 ± 3.57 (Control)	23.31 ± 3.50 (Control)					
Dai 2025 (22)	China	Case-control	198/40	26.31 ± 5.74 (SCH)	24.65 ± 5.67 (SCH)	Rotterdam	TSH > 4 mIU/L and normal T3/T4	Not performed	FSH, LH, estradiol, prolactin, total testosterone	Age(NR), BMI(NR), IR(NR), PCOS phenotype(NR), TAI(NR), Medication(NR), Infertility(NR), Cycle(M), Assay(M)
				26.75 ± 5.85 (Control)	20.75 ± 4.98 (Control)					
Acharya 2026 (23)	India	Cross-sectional	368/52	28.53 ± 3.29 (SCH)	27.56 ± 3.56 (SCH)	Rotterdam	TSH > 4.5 mIU/L and normal FT4	Not performed	FSH, LH, total testosterone	Age(NR), BMI(NR), IR(NR), PCOS phenotype(NR), TAI(NR), Medication(NR), Infertility(NR), Cycle(M), Assay(M)
				24.30 ± 2.95 (Control)	25.62 ± 2.65 (Control)					
Kamrul-Hasan 2022 (24)	Bangladesh	Cross-sectional	465/50	23.76 ± 5.27 (SCH)	27.39 ± 4.93 (SCH)	Rotterdam	TSH > 5 mIU/L and normal FT4	Not performed	prolactin, total testosterone	Age(NR), BMI(NR), IR(NR), PCOS phenotype(NR), TAI(NR), Medication(NR), Infertility(NR), Cycle(NR), Assay(M)
				22.37 ± 5.38 (Control)	26.53 ± 5.14 (Control)					
Shi 2024 (25)	China	Cross-sectional	133/58	28.03 ± 5.53 (SCH)	NA	Rotterdam	TSH > 4.5 mIU/L and normal FT4	Not performed	FSH, LH	Age(NR), BMI(NR), IR(A), PCOS phenotype(NR), TAI(NR), Medication(NR), Infertility(NR), Cycle(M), Assay(M)
				27.11 ± 4.75 (Control)						
Qasim 2025 (26)	Iraq	Cross-sectional	139/24	NA	28.74 ± 5.91 (SCH)	Rotterdam	TSH > 3.6 mIU/L and normal FT4	Not performed	FSH, LH, prolactin, total testosterone	Age(NR), BMI(NR), IR(NR), PCOS phenotype(NR), TAI(NR), Medication(NR), Infertility(NR), Cycle(M), Assay(M)
					27.93 ± 5.24 (Control)					
Gawron 2022 (27)	Poland	Retrospective cohort	367/98	24.15 ± 4.79 (SCH)	25.72 ± 6.48 (SCH)	Rotterdam	TSH > 2.5 mIU/L and normal FT4	Complete detection of TPOAb + TgAb	FSH, LH, estradiol, prolactin, total testosterone	Age(A), BMI(A), IR(A), PCOS phenotype(A), TAI(A), medication(NR), infertility(NR), Cycle(M), Assay(M)
				24.63 ± 5.51 (Control)	25.37 ± 6.67 (Control)					
de-Medeiros 2017 (28)	Brazil	Cross-sectional	493/31	28.73 ± 5.21 (SCH)	26.75 ± 6.18 (SCH)	Rotterdam	TSH ≥ 4.2 mIU/L and normal FT4	Not performed	FSH, LH, estradiol, prolactin, total testosterone	Age(M), BMI(M), IR(NR), PCOS phenotype(NR), TAI(NR), Medication(NR), Infertility(NR), Cycle(M), Assay(M)
				26.72 ± 5.38 (Control)	29.11 ± 6.74 (Control)					
Lu 2016 (29)	China	Case-control	196/92	25.86 ± 3.22 (SCH)	25.93 ± 4.73 (SCH)	Rotterdam	TSH ≥ 2.5 mIU/L and normal FT4	Not performed	FSH, LH, estradiol, prolactin, total testosterone	Age(M), BMI(NR), IR(NR), PCOS phenotype(NR), TAI(NR), Medication(NR), Infertility(NR), Cycle(M), Assay(M)
				25.30 ± 3.72 (Control)	24.27 ± 4.68 (Control)					
Yu 2016 (30)	China	Case-control	100/27	29.21 ± 5.9 (SCH)	32.70 ± 4.9 (SCH)	Rotterdam	TSH > 4.25 mIU/L and normal T3/T4	Not performed	FSH, LH, estradiol	Age(M), BMI(M), IR(NR), PCOS phenotype(NR), TAI(NR), Medication(NR), Infertility(NR), Cycle(M), Assay(M)
				26.37 ± 4.5 (control)	30.90 ± 5.1 (control)					

SCH, subclinical hypothyroidism; FT4, free thyroxine; T3, triiodothyronine; T4, thyroxine; TSH, thyroid-stimulating hormone; FSH, follicle-stimulating hormone; LH, luteinizing hormone; TPOAb, thyroid peroxidase antibody; TgAb, thyroglobulin antibody; NA, data not available; IQR, interquartile range; IR, insulin resistance; TAI, thyroid autoimmunity; Cycle, menstrual-cycle timing of blood sampling; Assay, standardized laboratory hormone assay methods. Abbreviations for confounding control status: M = Matched (baseline matching between SCH and euthyroid PCOS control groups); A = Adjusted (multivariable regression adjustment in statistical analyses); NR = Not reported/no matching or adjustment performed for the corresponding variable.

### 3.3 Quality evaluation of literature

**Figure 2:**
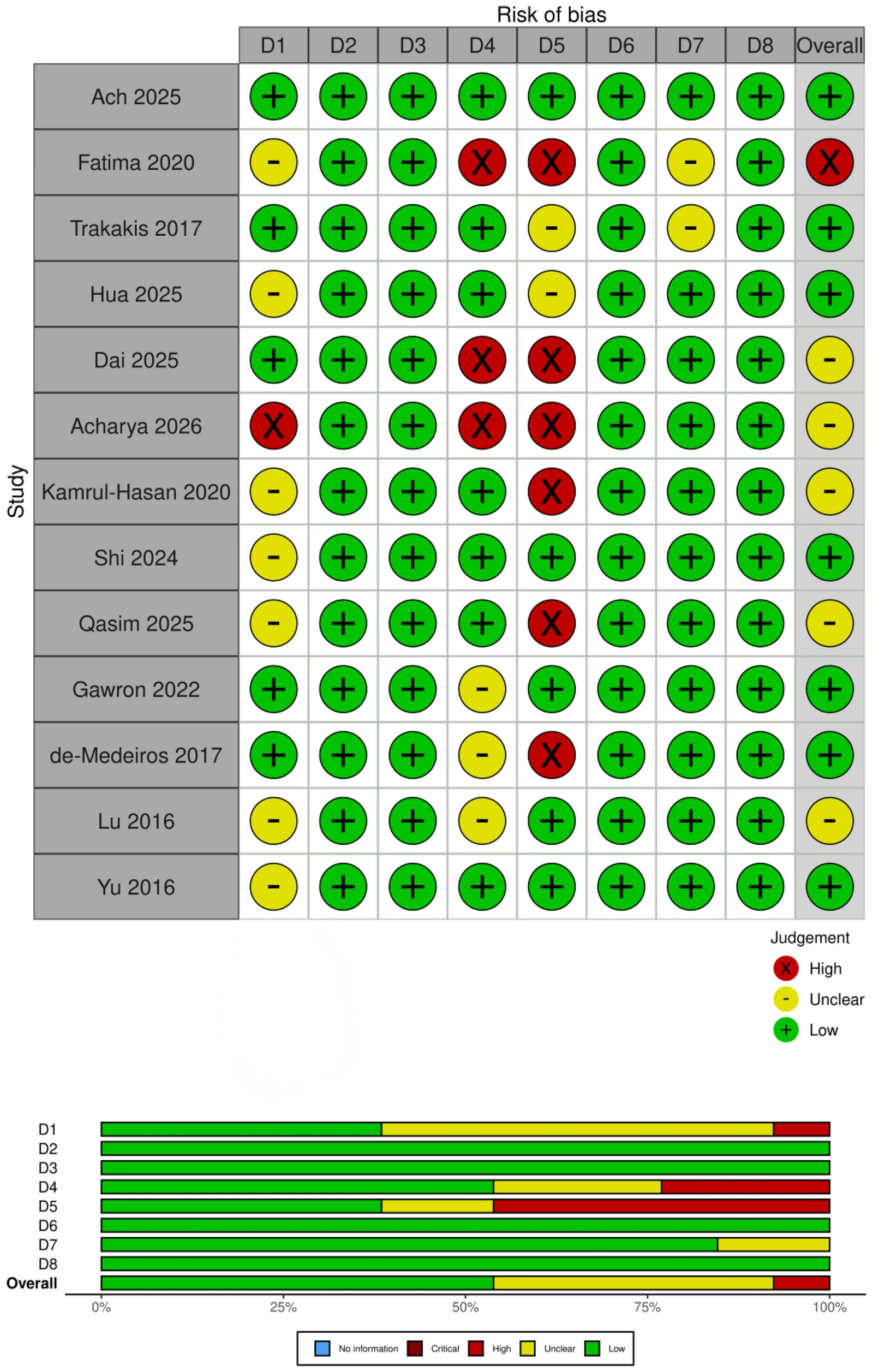
Domain-specific risk-of-bias assessment of the included observational studies. Definitions of eight evaluation domains: D1: Participant selection; D2: SCH definition and measurement; D3: PCOS diagnostic criteria; D4: Group comparability; D5: Confounder adjustment; D6: Hormone assay methods; D7: Timing of hormone measurement; D8: Completeness of outcome data.

**Table 3** and **Figure 2** present the domain-stratified risk-of-bias results of all included studies based on eight predefined evaluation dimensions, with original AHRQ total scores listed as supplementary evidence. Uniform low risk of bias was observed across all studies for D2 (SCH definition and measurement), D3 (PCOS diagnostic criteria), D6 (Hormone assay methods), and D8 (Completeness of outcome data), as all articles clearly reported unified diagnostic thresholds and complete laboratory testing information without missing core outcome data. The major methodological flaws were concentrated in D4 (Group comparability) and D5 (Confounder adjustment): several studies failed to balance baseline characteristics between groups or adjust for key confounding factors such as BMI and age, resulting in a high risk of bias. Moderate risk frequently appeared in D1 (Participant selection) and D7 (Timing of hormone measurement), caused by non-probability sampling and inadequate records of hormone blood collection cycles. Overall, seven studies had low overall bias risk, five had moderate risk, and the study by Fatima (19) was classified as high risk owing to dual high-risk ratings in group comparability and confounding control.

**Table 3:** Stratified risk-of-bias assessment by dimension of included studies.

Author	D1	D2	D3	D4	D5	D6	D7	D8	Original total score (AHRQ/NOS)	Overall risk of bias
Ach 2025 (18)	Low risk	Low risk	Low risk	Low risk	Low risk	Low risk	Low risk	Low risk	8/9	Low risk
Fatima 2020 (19)	Moderate risk	Low risk	Low risk	High risk	High risk	Low risk	Moderate risk	Low risk	6/9	High risk
Trakakis 2017 (20)	Low risk	Low risk	Low risk	Low risk	Moderate risk	Low risk	Moderate risk	Low risk	8/9	Low risk
Hua 2025 (21)	Moderate risk	Low risk	Low risk	Low risk	Moderate risk	Low risk	Low risk	Low risk	8/9	Low risk
Dai 2025 (22)	Low risk	Low risk	Low risk	High risk	High risk	Low risk	Low risk	Low risk	7/9	Moderate risk
Acharya 2026 (23)	High risk	Low risk	Low risk	High risk	High risk	Low risk	Low risk	Low risk	6/9	Moderate risk
Kamrul-Hasan 2020 (24)	Moderate risk	Low risk	Low risk	Low risk	High risk	Low risk	Moderate risk	Low risk	7/9	Moderate risk
Shi 2024 (25)	Moderate risk	Low risk	Low risk	Low risk	Low risk	Low risk	Low risk	Low risk	8/9	Low risk
Qasim 2025 (26)	Moderate risk	Low risk	Low risk	Low risk	High risk	Low risk	Low risk	Low risk	7/9	Moderate risk
Gawron 2022 (27)	Low risk	Low risk	Low risk	Moderate risk	Low risk	Low risk	Low risk	Low risk	8/9	Low risk
de-Medeiros 2017 (28)	Low risk	Low risk	Low risk	Moderate risk	High risk	Low risk	Low risk	Low risk	8/9	Low risk
Lu 2016 (29)	Moderate risk	Low risk	Low risk	Moderate risk	Low risk	Low risk	Low risk	Low risk	8/9	Moderate risk
Yu 2016 (30)	Moderate risk	Low risk	Low risk	Low risk	Low risk	Low risk	Low risk	Low risk	7/9	Low risk

Definitions of eight evaluation domains: D1: Participant selection; D2: SCH definition and measurement; D3: PCOS diagnostic criteria; D4: Group comparability; D5: Confounder adjustment; D6: Hormone assay methods; D7: Timing of hormone measurement; D8: Completeness of outcome data.

### 3.4 Comparison of serum hormone levels between PCOS patients with and without SCH

#### 3.4.1 FSH levels

**Figure 3:**
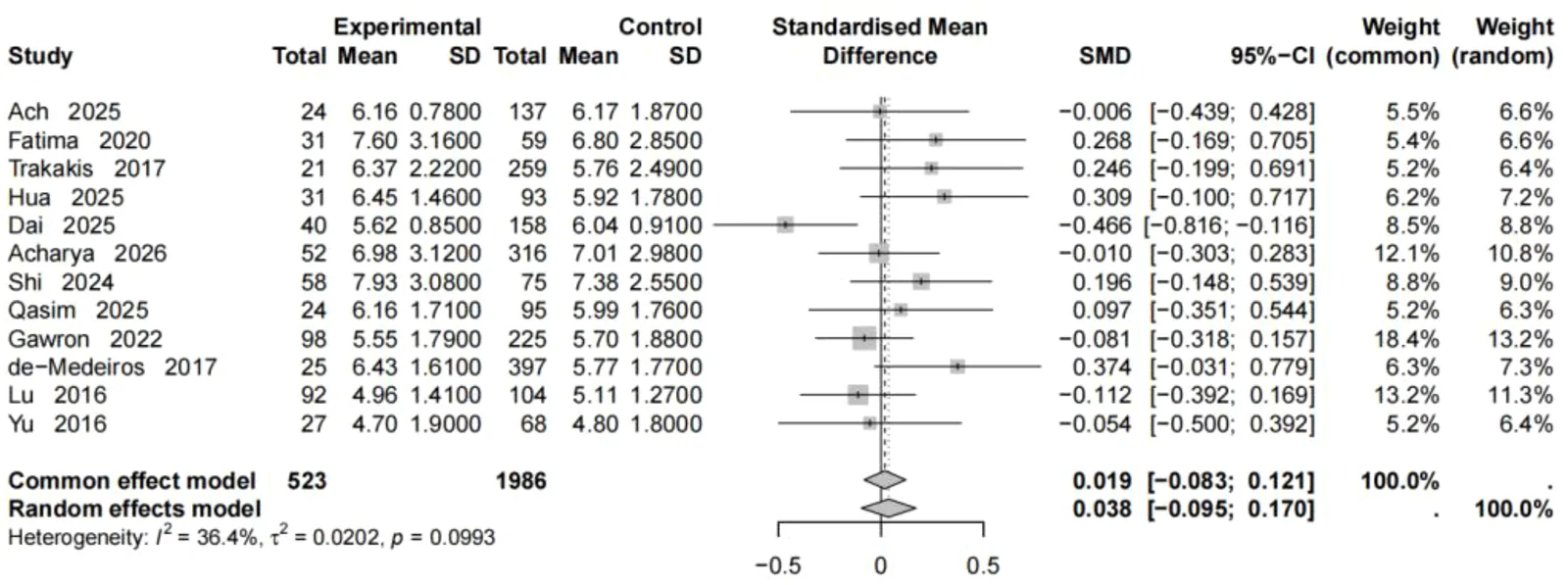
Meta-analysis of FSH levels in patients with PCOS with and without SCH.

**Figure 4:**
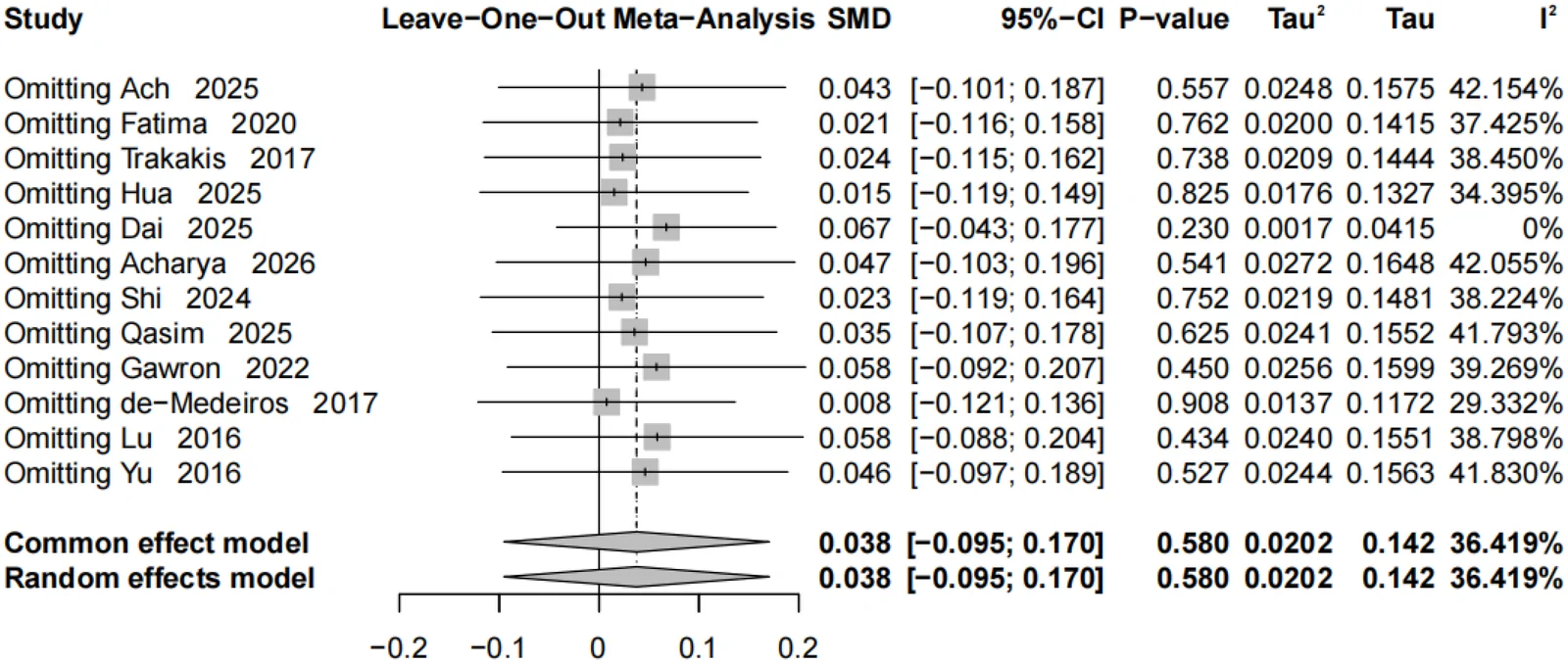
Leave-one-out sensitivity analysis of FSH levels.

A total of 12 studies (18–23, 25–30) were included. The analysis involved 523 PCOS patients with SCH and 1,986 euthyroid PCOS controls. The random-effects model revealed no significant difference in serum FSH levels between groups (SMD = 0.038, 95% CI: −0.095 to 0.170, 95% PI: −0.28 to 0.37, p = 0.581) (**Figure 3**). Moderate heterogeneity was detected (I² = 36.4%, Q-test p = 0.099; between-study variance τ² = 0.018). Leave-one-out analysis indicated the study by Dai et al. (22) as the main contributor to heterogeneity, which was resolved after its exclusion. The pooled outcome remained unchanged after sequential omission of individual studies (**Figure 4**). Sensitivity analysis based on the Hartung–Knapp-adjusted random-effects model generated consistent pooled effect sizes and overlapping CIs, with no change to the overall statistical inference. According to Cohen’s d criteria, the pooled SMD of 0.038 represents a negligible effect size (|SMD| < 0.2), suggesting that any observed difference in FSH concentrations is unlikely to be clinically meaningful for ovarian reserve evaluation based on current observational evidence.

#### 3.4.2 LH levels

**Figure 5:**
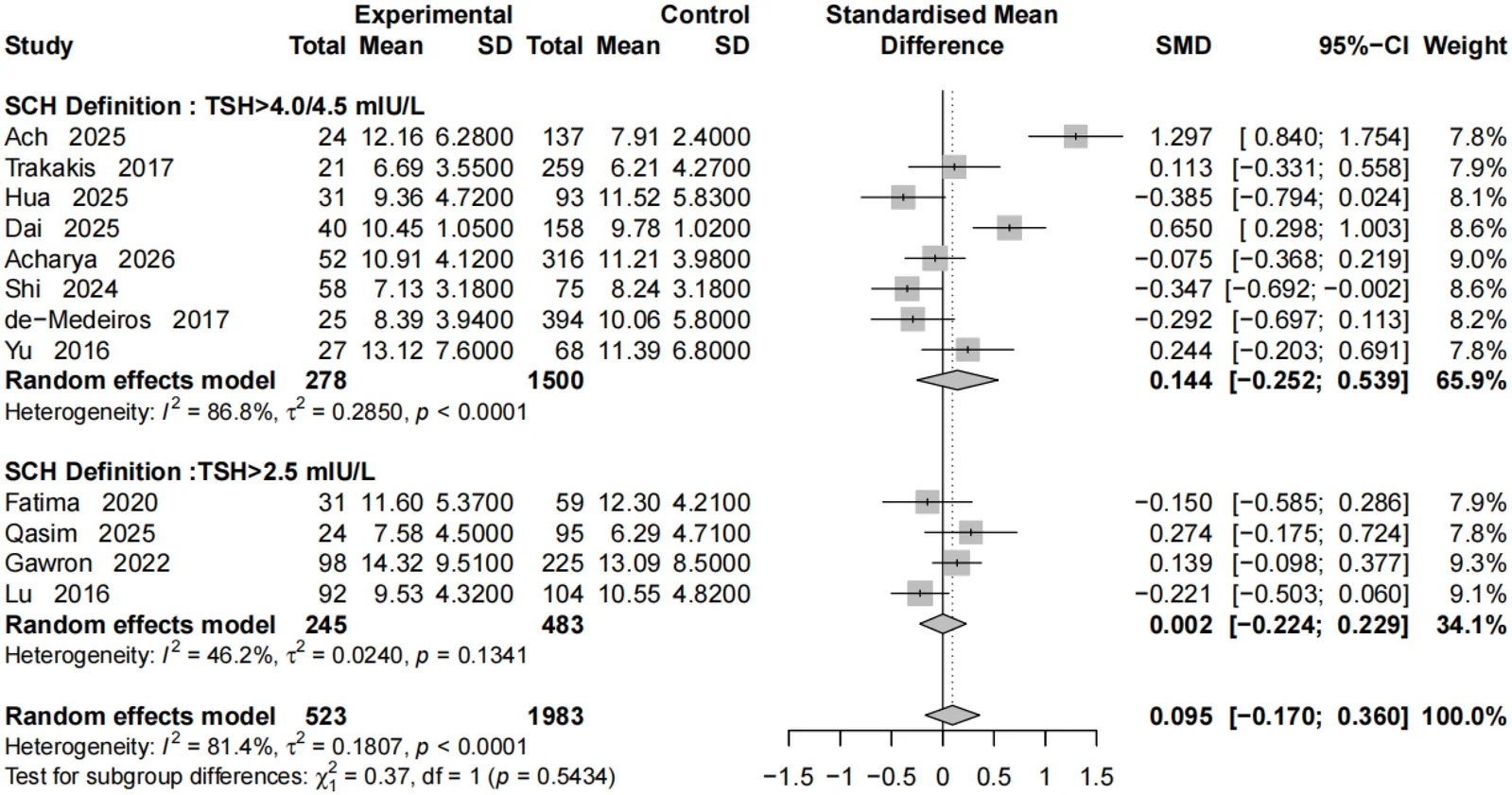
Meta-analysis of LH levels in patients with PCOS with and without SCH, stratified by TSH cutoff values.

Substantial heterogeneity was detected for LH (I² = 81.4%, p < 0.001, τ² = 0.1807). The pooled random-effects analysis showed no significant intergroup difference in LH (SMD = 0.095, 95% CI: −0.170 to 0.360, 95% PI: −0.88 to 1.08, p = 0.482). The wide PI and high heterogeneity suggest variable true effects across populations, limiting the clinical value of this pooled estimate. We performed subgroup analyses stratified by SCH TSH cutoffs to explore heterogeneity sources (**Figure 5**). No studies defined SCH using a combination of elevated TSH and positive thyroid autoantibodies, so only two subgroups were pooled: seven studies using a conventional cutoff (> 4.0/4.5 mIU/L, SMD = 0.144, 95% CI: −0.252 to 0.539, I² = 86.8%, p < 0.001) and four using a low preconception cutoff (> 2.5 mIU/L, SMD = 0.002, 95% CI: −0.224 to 0.229, I² = 46.2%, p = 0.1341). Subgroup interaction was non-significant (χ² = 0.37, p = 0.5434), yet divergent subgroup I² values imply that inconsistent TSH thresholds partially drive overall heterogeneity (18–23, 25–30). Small subgroup sample sizes and broad PIs cannot rule out distinct hormonal effects across SCH cutoff groups. Hartung–Knapp sensitivity analysis yielded consistent pooled estimates without altering statistical inference. The pooled SMD of 0.095 represents a negligible effect (|SMD| < 0.2); based on current observational data, this magnitude of difference is unlikely to reflect clinically relevant pituitary-gonadal axis changes in women with PCOS.

#### 3.4.3 Estradiol levels

**Figure 6:**
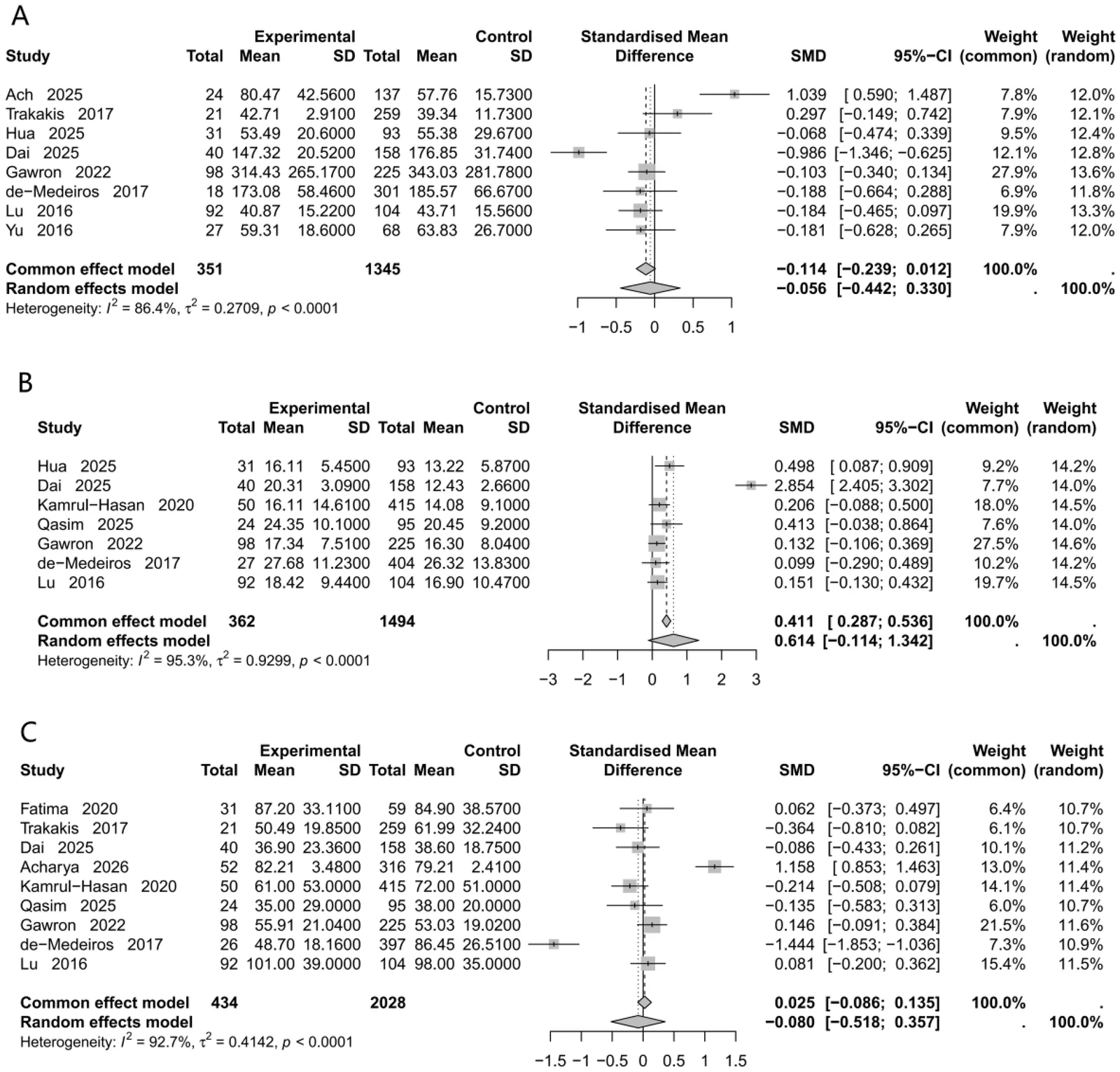
Meta-analysis of estradiol **(A)**, prolactin **(B)**, and total testosterone **(C)** levels in patients with PCOS with and without SCH.

Eight studies (18, 20–22, 27–30) including 351 PCOS patients with SCH and 1,345 euthyroid PCOS controls were pooled for estradiol analysis. Random-effects meta-analysis detected no significant intergroup difference (SMD = −0.056, 95% CI: −0.442 to 0.330, 95% PI: −1.37 to 1.24, p = 0.762; I² = 86.4%, τ² = 0.271, p < 0.001; **Figure 6A**). The extremely wide PI and severe between-study heterogeneity restricts clinical interpretation, so this null finding cannot definitively exclude a true association.

**Figure 7:**
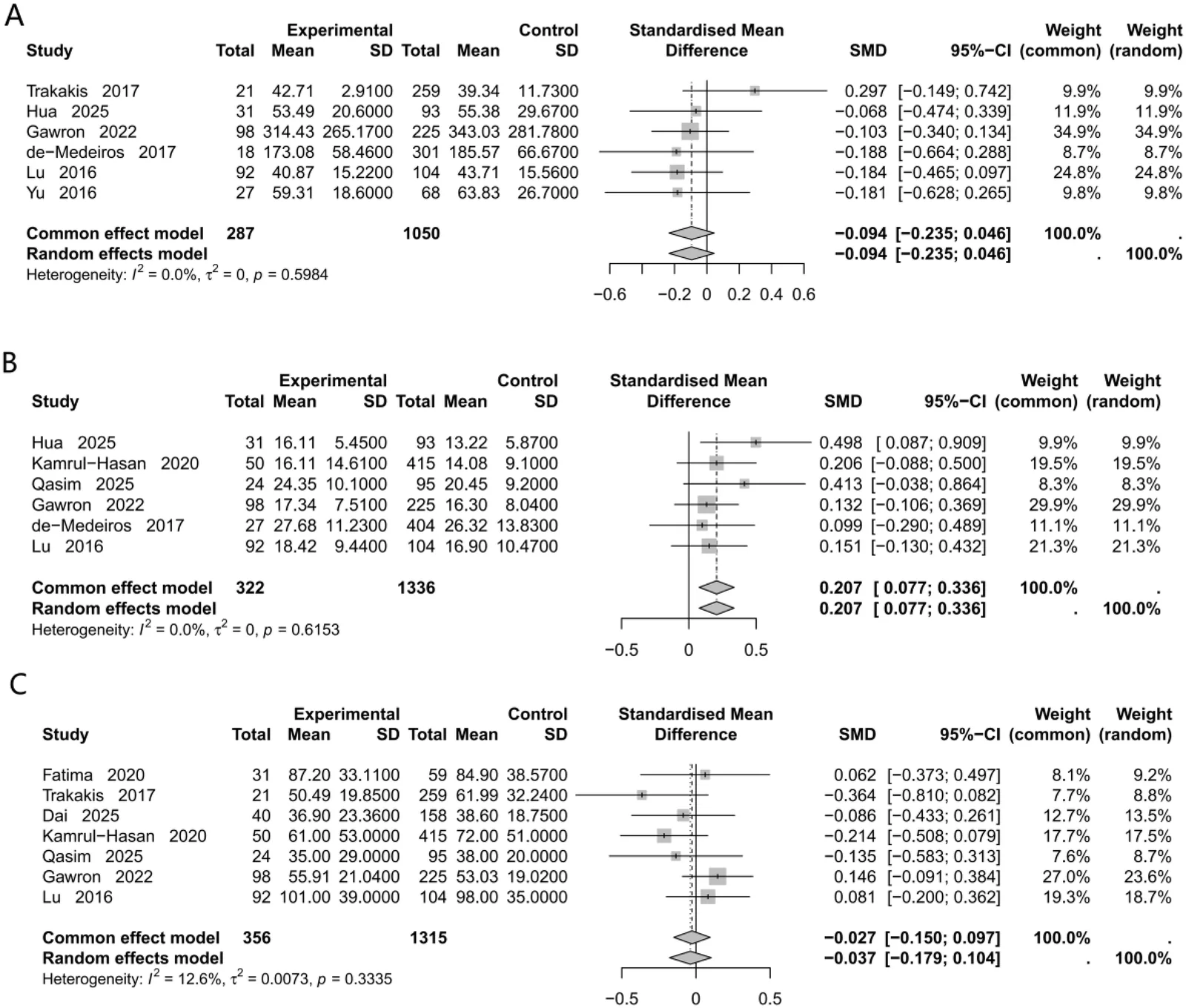
Leave-one-out sensitivity analysis of estradiol **(A)**, prolactin **(B)**, and total testosterone **(C)** levels in patients with PCOS with and without SCH.

Leave-one-out analysis identified Ach et al. (18) and Dai et al. (22) as the main contributors to heterogeneity; their removal eliminated heterogeneity (I² = 0%) without shifting the non-significant pooled result, and all leave-one-out CIs crossed zero (**Figure 7A**). Hartung–Knapp sensitivity analysis yielded consistent effect sizes and unchanged overall inference. The pooled SMD of −0.056 falls well below the 0.2 threshold for a clinically meaningful effect, suggesting that current observational evidence does not support an association between SCH and discernible changes in estradiol concentrations in women with PCOS.

#### 3.4.4 Prolactin levels

Seven studies (21, 22, 24, 26–29) comprising 362 PCOS patients with SCH and 1,494 euthyroid PCOS controls were pooled for prolactin analysis. The primary random-effects model showed no significant intergroup difference (SMD = 0.614, 95% CI: −0.114 to 1.342, 95% PI: −1.81 to 2.85, p = 0.165 for the pooled effect; τ² = 0.923, I² = 95.3%, p < 0.001 for the Q-test; **Figure 6B**). Extremely high heterogeneity and a broad PI greatly limit the reliability of this pooled estimate, so the null finding cannot fully exclude a true SCH-related prolactin effect. Leave-one-out analysis identified Dai et al. (22) as the major source of heterogeneity; its exclusion produced a significant small pooled effect (SMD = 0.207, 95% CI: 0.077 to 0.336), with heterogeneity fully eliminated (I² = 0%, τ² = 0, Q-test p = 0.615; **Figure 7B**). However, this outlier had no identifiable methodological flaws justifying *post-hoc* removal, and our PROSPERO protocol did not predefine criteria for omitting influential studies. Accordingly, this *post-hoc* significant result is exploratory only, and the full-sample pooled analysis remains our primary inference for prolactin. Hartung–Knapp sensitivity analysis produced stable overall effect estimates with no change in statistical conclusions. According to Cohen’s d thresholds, the original SMD of 0.614 reflects a moderate statistical effect, but it is heavily skewed by extreme heterogeneity and the single outlier. After outlier exclusion, the SMD fell to 0.207, representing a small effect whose clinical relevance cannot be determined from hormone level data alone.

#### 3.4.5 Total testosterone levels

Nine studies (19, 20, 22–24, 26–29) including 434 PCOS patients with SCH and 2,028 euthyroid PCOS controls were analyzed for total testosterone. The random-effects model revealed no significant intergroup difference (SMD = 0.08, 95% CI: −0.158 to 0.357, 95% PI: −1.04 to 1.09, p = 0.882; τ² = 0.414, I² = 92.7%, Q-test p < 0.001; **Figure 6C**). Severe heterogeneity and a wide PI weaken causal inference, so this null result does not definitively rule out a SCH-related testosterone effect. Leave-one-out analysis maintained consistent non-significant findings across all omissions (**Figure 7C**). Acharya et al. (23) and de-Medeiros et al. (28) drove most heterogeneity; their removal yielded a still non-significant pooled estimate (SMD = −0.037, 95% CI: −0.179 to 0.104, p > 0.05, I² = 12.6%) with unchanged effect direction. Hartung–Knapp sensitivity analysis validated stable primary results. The SMD of 0.08 represents a negligible effect size, suggesting that the observed testosterone difference between groups is unlikely to affect clinical evaluation of hyperandrogenism in PCOS based on current observational evidence.

### 3.5 Sensitivity analysis based on risk of bias

Sensitivity analyses excluding high-risk studies and studies with converted hormone data yielded pooled estimates directionally consistent with the primary analyses for all five hormones, with no outcome changing from non-significant to significant (**Supplementary Table 3** and **S4**). For prolactin, restriction to low-risk studies markedly reduced heterogeneity (I² = 22.7%) and suggested a potential, albeit non-significant, increase associated with SCH. Furthermore, the estradiol estimate shifted from near-null (SMD = −0.056) to a more pronounced negative value (SMD = −0.326, 95% CI: −0.654 to 0.002) after excluding converted-data studies, suggesting that these studies may have introduced noise toward the null; however, this finding remained non-significant and was based on a reduced sample.

### 3.6 Publication bias

**Figure 8:**
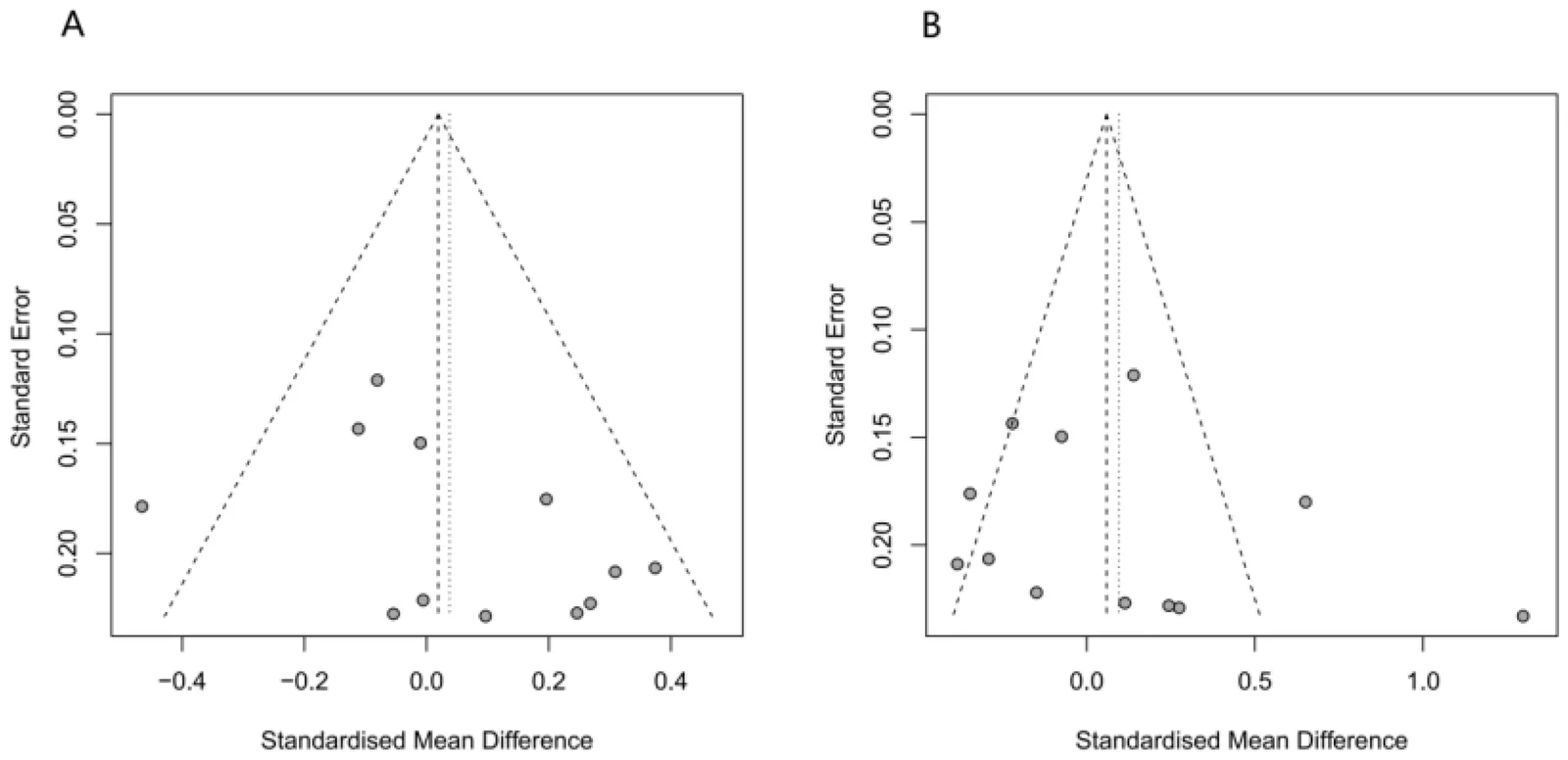
Funnel plots for assessing publication bias in the meta-analyses of FSH **(A)** and LH **(B)**.

Funnel plots and Egger’s test were performed for FSH and LH, for which 10 or more studies were included. Funnel plots for both outcomes were approximately symmetric (**Figure 8**), and Egger’s test yielded non-significant p-values (FSH: p = 0.410; LH: p = 0.478), indicating no evidence of substantial publication bias for these two outcomes. For estradiol, prolactin, and total testosterone, which each included fewer than 10 studies, Egger’s test and Begg’s test were conducted for descriptive purposes only. Statistical power to detect publication bias is known to be very limited with fewer than 10 studies, so no definitive conclusion regarding the absence of publication bias can be drawn for these outcomes. The Egger’s test p-values were 0.540 for estradiol, 0.181 for prolactin, and 0.274 for total testosterone. Furthermore, Begg’s test reached statistical significance for prolactin (p = 0.024), which may indicate potential small-study effects or reporting bias for this outcome. However, this finding should be interpreted cautiously given the small number of included studies and limited statistical power.

### 3.7 GRADE certainty of evidence assessment

We graded the certainty of the evidence for all reproductive hormone outcomes using the GRADE-like framework for observational meta-analyses, evaluating risk of bias, inconsistency, indirectness, imprecision, and publication bias. Only FSH yielded low-certainty evidence, while LH, estradiol, prolactin, and total testosterone were rated as very-low-certainty evidence. Full grading details are shown in **Supplementary Table 5**.

## 4 Discussion

This study aimed to examine the associations between SCH and key reproductive hormone levels in patients with PCOS using a systematic review and meta-analysis of observational studies. Contrary to what was expected from some recent clinical observational studies, the overall pooled analyses did not identify consistent, statistically significant differences in major reproductive hormone levels between patients with PCOS with and without SCH. Given the substantial between-study heterogeneity observed for multiple outcomes and the observational nature of the included studies, this finding does not definitively rule out subtle hormonal effects of SCH. Instead, the current observational evidence does not demonstrate consistent associations between SCH and core reproductive hormone profiles at the pooled population level. Metabolic parameters were more frequently reported as affected than reproductive hormones in the included studies, although this comparison was not formally evaluated.

In the present analysis, no statistically significant overall differences in FSH and LH levels were identified between PCOS patients with and without SCH. These results are broadly consistent with the findings of Pergialiotis et al. (8) and suggest that SCH is not associated with large disruptions to the pituitary-gonadal axis in PCOS populations based on available observational data. This pattern is consistent with observations that metabolic disturbances are more commonly reported in association with SCH than reproductive hormone alterations in PCOS (31, 32). Physiologically, both pituitary thyrotrophs and gonadotrophs are regulated by the hypothalamus, yet they differ in sensitivity to hormonal feedback (33). In the SCH state, elevated TSH reflects mild thyroid hormone insufficiency, while free thyroxine typically remains within the normal range. One speculative explanation for the absence of observed associations is that normal FT4 levels might sustain steady basal regulation of FSH and LH by the hypothalamus and pituitary (12). Alternatively, the elevated LH/FSH ratio typically observed in PCOS has been primarily attributed to follicular arrest and abnormal hypothalamic feedback related to granulosa cell dysfunction (34); whether mild thyroid imbalance contributes independently remains uncertain given the observational design of the available evidence. Whether correcting mild SCH improves ovulatory dysfunction in PCOS remains unaddressed by current evidence, as this meta-analysis evaluated hormone levels only and did not assess ovulation or other clinical endpoints.

Although hyperandrogenism is a core feature of PCOS, thyroid hormones may indirectly influence androgen levels by regulating hepatic SHBG synthesis or adrenal androgen secretion (10). However, our pooled analyses do not provide consistent evidence to support this hypothesis. The overall pooled estimate did not show a statistically significant elevation of total testosterone levels in PCOS patients with SCH, but very high between-study heterogeneity limits the strength of this conclusion. This finding should be interpreted cautiously given the observational nature of included studies. Hyperandrogenism in PCOS has been mainly attributed to intrinsic enzymatic abnormalities in ovarian theca cells, such as CYP17 overexpression, or insulin-related metabolic disorders (35). The extent to which mild thyroid dysfunction may independently contribute to androgen levels in PCOS cannot be determined from the observational evidence currently available. Previous individual studies suggested that SCH might raise estradiol levels (18). Our meta-analysis showed that this association did not remain statistically significant at the overall pooled population level, but high heterogeneity and inconsistent effect directions across studies indicate that the relationship remains uncertain. Sensitivity analyses excluding studies with converted data yielded a more pronounced negative point estimate for estradiol (SMD = −0.326, 95% CI: −0.654 to 0.002), raising the possibility that distributional transformations in the excluded studies introduced noise toward the null; however, this finding is exploratory, non-significant, and based on a reduced sample, so no firm conclusion can be drawn.

Nevertheless, significant statistical heterogeneity (I² > 50%) was observed in analyses of LH, estradiol, and prolactin. Subgroup and sensitivity analyses identified major sources of heterogeneity. Variable diagnostic criteria for SCH represented a key contributor to heterogeneity in the LH analysis. Included studies adopted TSH cutoffs ranging from above 2.5 to 5.0 mIU/L. Different thresholds reflect distinct degrees of thyroid compensatory status. A TSH cutoff of > 2.5 mIU/L is often applied in pregnant or preconception populations as a more sensitive physiological standard, while TSH > 4.5 mIU/L indicates a more pronounced hypothyroid tendency (36). Such inconsistent definitions may have contributed to differences in baseline biological characteristics and are likely to have introduced clinical heterogeneity.

Subgroup analysis indicated that heterogeneity was markedly higher within the conventional TSH > 4.0/4.5 mIU/L group (I² = 86.8%) compared with the low 2.5 mIU/L cutoff group (I² = 46.2%). Although formal testing detected no statistically significant difference in effect sizes between the two subgroups (p = 0.5434), this statistical null result does not negate clinically important differences between these two SCH cohorts. Merging patients defined by disparate TSH thresholds into a single uniform SCH group oversimplifies thyroid–ovarian endocrine interactions and risks overgeneralizing our overall findings. This aligns with the suggestion that SCH should not be interpreted as a homogeneous exposure. In addition to variable TSH cutoffs, thyroid autoimmunity status represents another potentially important source of clinical heterogeneity that could not be explored quantitatively in this meta-analysis. SCH with positive thyroid autoantibodies (typically caused by Hashimoto’s thyroiditis) and SCH without autoimmunity represent distinct clinical phenotypes. Autoimmune thyroid injury has been associated with chronic low-grade inflammation and immune dysregulation, which may interfere with ovarian steroidogenesis and pituitary-gonadal axis function, whereas non-autoimmune mild TSH elevation may primarily reflect subtle thyroid compensatory dysfunction with potentially minimal inflammatory impact. These proposed mechanisms remain hypothetical in the context of PCOS and require interventional or prospective longitudinal studies for validation. The single available stratified study (23) suggested that PCOS patients with autoimmune SCH had more pronounced metabolic disturbances, but evidence regarding its independent impact on core reproductive hormones was limited. Due to insufficient primary data, we could not verify whether thyroid autoimmunity drives divergent hormonal effects across studies. This unassessed confounding factor may partially explain the high heterogeneity observed in LH, estradiol and prolactin analyses.

Regional and ethnic disparities also played an important role. This meta-analysis enrolled populations from China, Greece, Tunisia, and other regions. Differences in iodine nutrition, genetic background, and exposure to environmental endocrine disruptors may directly modulate crosstalk between the thyroid and ovarian axes (37). Inadequate control of confounding factors further amplified heterogeneity. Obesity and insulin resistance are common comorbidities in PCOS patients. Insulin resistance independently regulates LH secretion and ovarian androgen production. Failure to match for BMI or adjust for insulin levels may mask the true hormonal effects of SCH (38, 39).

Interpretation of prolactin, LH, estradiol and total testosterone requires caution. A significant prolactin difference emerged after *post-hoc* exclusion of Dai et al. (22), yet this study had no identifiable methodological or clinical features justifying preplanned removal, and our PROSPERO protocol did not set criteria for omitting influential studies. All leave-one-out analyses across the four hormones serve only to explore heterogeneity and yield hypothesis-generating signals that cannot override the primary pooled estimates from the full dataset. Hypothyroidism can theoretically stimulate pituitary prolactin secretion via thyrotropin-releasing hormone (TRH). In PCOS patients, however, this biological effect may be masked by prevalent hyperleptinemia or chronic inflammation inherent to PCOS, leading to unstable pooled outcomes (40, 41).

Bias-stratified sensitivity analyses illustrated how methodological quality affected results for each hormonal indicator. For FSH, moderate- and high-bias studies contributed substantially to between-study heterogeneity, while analyses across different quality subgroups all failed to detect a measurable association between SCH and FSH levels. For LH, estradiol, and total testosterone, substantial between-study heterogeneity remained evident even when only low-risk studies were analyzed, suggesting inconsistent findings across publications were driven by clinical characteristics rather than flawed study design. Analyses stratified by risk of bias did not yield evidence of an association between SCH and these three hormones. For prolactin, studies with moderate risk of bias that lacked adjustment for metabolic confounders increased overall heterogeneity, which may obscure a small elevation of prolactin linked to SCH that was observed in low-risk, well-adjusted studies. Taken together, methodological limitations primarily altered the degree of between-study heterogeneity. Analyses restricted to low-risk studies did not identify hormonal associations between SCH and reproductive endocrine parameters among patients with PCOS.

All results should be interpreted cautiously given the observational nature of the included studies, which allow only descriptions of associations rather than causal inference. This meta-analysis showed no consistent differences in reproductive hormone levels between patients with PCOS with and without SCH, while coexisting SCH was frequently accompanied by more severe obesity and glucose metabolism disorders (42, 43). The core PCOS marker of an elevated LH/FSH ratio remained unchanged across thyroid status. Importantly, we analyzed only hormone concentrations without clinical reproductive endpoints such as ovulation, pregnancy, and miscarriage, so we cannot judge the therapeutic value of levothyroxine for these outcomes. Regular metabolic screening is recommended for this group at high metabolic risk, but thyroid evaluation for individual patients should therefore not be abandoned.

For clinical practice, SCH does not affect the Rotterdam diagnostic criteria for PCOS, and thyroid function should not interfere with PCOS diagnosis. The small effect sizes of SCH-related hormonal changes cannot independently guide fertility treatment decisions, though metabolic monitoring is still necessary for women with PCOS and SCH. Current hormone-based evidence is insufficient to adjust thyroid screening guidelines for PCOS patients, and routine thyroid testing should follow existing recommendations. Since reproductive and pregnancy outcomes were not assessed in this review, whether levothyroxine treatment can improve endocrine and fertility outcomes remains unclear and requires further investigation in randomized controlled trials.

This study has several limitations. First, variable TSH cutoffs for SCH (2.5–5.0 mIU/L) and insufficient thyroid autoantibody data limited stratified analyses and generalizability. Second, all studies were observational; most matched only age and BMI without multivariable adjustment for insulin resistance, PCOS phenotype, thyroid autoimmunity, medication use, infertility status, or menstrual phase, leaving residual confounding. Third, heterogeneity in hormone assays and non-standardized menstrual-cycle timing across laboratories introduced measurement variability. Fourth, inclusion of English-only articles and exclusion of gray literature introduced a risk of language bias. Fifth, protocol registration followed literature retrieval, and publication bias tests were underpowered for estradiol, prolactin, and testosterone. Sixth, GRADE certainty was low for FSH and very low for the remaining hormones. These limitations preclude causal inference.

## 5 Conclusions

Current observational evidence does not demonstrate a consistent difference in FSH, LH, estradiol, or total testosterone levels between patients with PCOS and SCH and euthyroid PCOS controls. The evidence for prolactin is uncertain because of high heterogeneity and sensitivity to influential studies. Given the heterogeneity in SCH definitions, residual confounding, and the absence of interventional or reproductive-outcome data, these findings should be interpreted with caution. Further well-designed prospective studies, ideally stratified by thyroid antibody status, BMI, insulin resistance, and PCOS phenotype, are needed to clarify whether SCH independently affects reproductive endocrine function and clinically relevant reproductive outcomes in PCOS.

## Data Availability

The original contributions presented in the study are included in the article/**Supplementary Material**. Further inquiries can be directed to the corresponding author.
