# Supplementary material for: The impact of subclinical hypothyroidism on core reproductive hormones and ovarian endocrine status in patients with polycystic ovary syndrome: a systematic review and meta-analysis
Source: Front Endocrinol (Lausanne). 2026 Sep 11;17:1893428. doi: 10.3389/fendo.2026.1893428 (PMC13612295; doi:10.3389/fendo.2026.1893428)
Supplement: Supplementary file 1 [file DataSheet1.docx]

Supplementary Material

# 1 Supplementary Tables

Table S1 Search strategies for the four databases

| Database | Search strategy |
| --- | --- |
| PubMed | ("Polycystic Ovary Syndrome"[Mesh] OR "Polycystic Ovary Syndromes"[Title/Abstract] OR "PCOS"[Title/Abstract] OR "Stein-Leventhal Syndrome"[Title/Abstract]) AND ("Thyroid Function Tests"[Mesh] OR "Hypothyroidism"[Mesh] OR "Subclinical Hypothyroidism"[Title/Abstract] OR "SCH"[Title/Abstract] OR "Subclinical Thyroid"[Title/Abstract] OR "Thyroid Function"[Title/Abstract] OR "TSH"[Title/Abstract] OR "Thyrotropin"[Title/Abstract]) |
| Web of Science | #1 (Polycystic Ovary Syndrome*) OR (PCOS) OR (Stein-Leventhal Syndrome)  #2 (Subclinical Hypothyroidism) OR (SCH) OR (Subclinical Thyroid) OR (Thyroid Function*) OR (TSH) OR (Thyrotropin)  #3 #1 AND #2 |
| Embase | ('polycystic ovary syndrome'/exp OR 'polycystic ovary syndrome':ti,ab,kw OR 'pcos':ti,ab,kw OR 'stein leventhal syndrome':ti,ab,kw) AND ('subclinical hypothyroidism'/exp OR 'subclinical hypothyroidism':ti,ab,kw OR 'sch':ti,ab,kw OR 'subclinical thyroid':ti,ab,kw OR 'thyroid function':ti,ab,kw OR 'thyrotropin'/exp OR 'tsh':ti,ab,kw) |
| Cochrane Library | #1 polycystic ovary syndrome* OR "PCOS" OR "stein-leventhal syndrome*"  #2 subclinical hypothyroidism OR "SCH" OR "subclinical thyroid" OR "thyroid function*" OR "TSH" OR "thyrotropin"  #3 #1 AND #2 |

Table S2 Seven supplied reports with unavailable or unusable quantitative outcome data

| First author | Year | Country | Topic relevance | Specific reason for exclusion or reassessment |
| --- | --- | --- | --- | --- |
| Rosivari PS | 2022 | India | PCOS+SCH; total testosterone mentioned | Group-specific sample size, mean, and dispersion for an eligible PCOS+SCH versus euthyroid PCOS total-testosterone contrast could not be verified in the supplied report. Reassess before submission. |
| Raj | 2021 | Pakistan | PCOS+SCH comparator | No extractable group-specific summary statistics for any eligible hormone outcome in the supplied report. |
| Enzevaei | 2014 | Iran | PCOS+SCH | Free testosterone was reported, but an eligible total-testosterone construct and extractable group summaries for FSH, LH, estradiol, or prolactin were unavailable. |
| Bedaiwy MA | 2018 | USA | PCOS+SCH comparator | No extractable group-specific numerical summary for an eligible hormone outcome was available in the supplied report. |
| Huang | 2014 | China | PCOS+SCH | Reported lipid profiles and an LH/FSH ratio, but no extractable group summaries for the prespecified hormone outcomes. |
| Krysiak R | 2014 | Poland | PCOS+SCH intervention setting | Concurrent treatment/co-treatment prevented extraction of an eligible untreated PCOS+SCH versus euthyroid PCOS hormone contrast. |
| Zhang B | 2018 | China | Thyroid/obesity study | No separate eligible PCOS subgroup with a PCOS+SCH versus euthyroid PCOS hormone comparison. |

Note: This table describes the seven excluded reports supplied in the revision archive. It is not a complete citation-level log of the 272 full-text exclusions. The complete log, including one specific reason for every excluded report, must be appended from the original screening records before submission.

Table S3. Primary, transformation, source-integrity, and thyroid-autoimmunity sensitivity analyses

| Outcome | Analysis set | k | Hedges g (95% CI) | I^2^ (%) | 95% PI |
| --- | --- | --- | --- | --- | --- |
| FSH | Primary | 12 | 0.04 (-0.10 to 0.19) | 34.1 | -0.28 to 0.37 |
| FSH | Exclude converted records | 9 | 0.03 (-0.16 to 0.23) | 48.7 | -0.40 to 0.47 |
| FSH | Exclude source-integrity-flagged records | 5 | 0.04 (-0.19 to 0.27) | 17.7 | -0.23 to 0.31 |
| FSH | Exclude Acharya, Dai, and Shi | 9 | 0.08 (-0.07 to 0.22) | 0.0 | -0.10 to 0.26 |
| FSH | Gawron ATA-negative replacement | 12 | 0.04 (-0.10 to 0.19) | 34.2 | -0.29 to 0.37 |
| LH | Primary | 12 | 0.10 (-0.20 to 0.40) | 81.8 | -0.88 to 1.08 |
| LH | Exclude converted records | 9 | -0.04 (-0.30 to 0.22) | 73.1 | -0.75 to 0.68 |
| LH | Exclude source-integrity-flagged records | 4 | -0.04 (-0.45 to 0.37) | 61.7 | -0.78 to 0.70 |
| LH | Exclude Acharya, Dai, and Shi | 9 | 0.11 (-0.27 to 0.49) | 81.6 | -0.98 to 1.20 |
| LH | Gawron ATA-negative replacement | 12 | 0.09 (-0.21 to 0.40) | 81.5 | -0.89 to 1.07 |
| Estradiol | Primary | 8 | -0.06 (-0.53 to 0.40) | 86.2 | -1.37 to 1.24 |
| Estradiol | Exclude converted records | 6 | -0.28 (-0.65 to 0.09) | 73.5 | -1.13 to 0.57 |
| Estradiol | Exclude source-integrity-flagged records | 4 | 0.13 (-0.81 to 1.06) | 87.3 | -1.84 to 2.10 |
| Estradiol | Exclude Acharya, Dai, and Shi | 7 | 0.07 (-0.34 to 0.47) | 76.6 | -0.94 to 1.08 |
| Estradiol | Gawron ATA-negative replacement | 8 | -0.06 (-0.53 to 0.40) | 86.2 | -1.37 to 1.24 |
| Prolactin | Primary | 8 | 0.52 (-0.27 to 1.32) | 94.7 | -1.81 to 2.85 |
| Prolactin | Exclude converted records | 5 | 0.74 (-0.73 to 2.21) | 96.8 | -2.81 to 4.29 |
| Prolactin | Exclude source-integrity-flagged records | 3 | 0.13 (-0.22 to 0.48) | 0.0 | -0.22 to 0.48 |
| Prolactin | Exclude Acharya, Dai, and Shi | 7 | 0.18 (0.02 to 0.33) | 0.0 | 0.02 to 0.33 |
| Prolactin | Gawron ATA-negative replacement | 8 | 0.52 (-0.27 to 1.32) | 94.7 | -1.81 to 2.85 |
| Total testosterone | Primary | 9 | 0.02 (-0.34 to 0.38) | 87.3 | -1.04 to 1.09 |
| Total testosterone | Exclude converted records | 6 | 0.16 (-0.38 to 0.70) | 89.6 | -1.20 to 1.51 |
| Total testosterone | Exclude source-integrity-flagged records | 3 | -0.01 (-0.67 to 0.65) | 61.1 | -1.07 to 1.05 |
| Total testosterone | Exclude Acharya, Dai, and Shi | 7 | -0.10 (-0.32 to 0.12) | 47.1 | -0.55 to 0.35 |
| Total testosterone | Include ambiguous Fatima testosterone (scenario) | 10 | 0.03 (-0.29 to 0.35) | 85.7 | -0.95 to 1.00 |
| Total testosterone | Gawron ATA-negative replacement | 9 | 0.02 (-0.34 to 0.38) | 87.3 | -1.04 to 1.09 |

Note: All models are random effects with REML tau² and safeguarded Hartung-Knapp confidence intervals. The source-integrity sensitivity is outcome specific and is not a low-risk-only analysis; every pooled contrast remains unadjusted.

Table S4 TSH-cutoff subgroup analyses

| Outcome | Subgroup | k | Hedges g (95% CI) | I^2^(%) | 95% PI | Interaction P |
| --- | --- | --- | --- | --- | --- | --- |
| FSH | Lower cutoff/high-normal (2.5-3.6 mIU/L) | 4 | 0.00 (-0.24 to 0.25) | 0.0 | -0.24 to 0.25 | 0.202 |
| FSH | Conventional cutoff (4.0-5.0 mIU/L) | 7 | 0.14 (-0.04 to 0.32) | 0.0 | -0.04 to 0.32 | 0.202 |
| LH | Lower cutoff/high-normal (2.5-3.6 mIU/L) | 4 | 0.02 (-0.38 to 0.43) | 57.4 | -0.69 to 0.74 | 0.863 |
| LH | Conventional cutoff (4.0-5.0 mIU/L) | 7 | 0.07 (-0.47 to 0.60) | 85.7 | -1.34 to 1.48 | 0.863 |

Table S5 GRADE-adapted summary of findings

| Outcome | Studies / participants | Hedges g (95% CI) | 95% PI | Reasons for downgrading | Publication-bias assessment | Certainty |
| --- | --- | --- | --- | --- | --- | --- |
| FSH | 12/2553 | 0.04 (95% CI -0.10 to 0.19) | -0.28 to 0.37 | RoB serious (unadjusted contrasts; source concerns); inconsistency not serious; indirectness serious (heterogeneous SCH definitions; limited clinical applicability); imprecision serious (CI and/or PI span different directions). | No clear signal detected; cannot be excluded. | Very low |
| LH | 12/2552 | 0.10 (95% CI -0.20 to 0.40) | -0.88 to 1.08 | RoB serious (unadjusted contrasts; source concerns); inconsistency serious; indirectness serious (heterogeneous SCH definitions; limited clinical applicability); imprecision serious (CI and/or PI span different directions). | No clear signal detected; cannot be excluded. | Very low |
| Estradiol | 8/1740 | -0.06 (95% CI -0.53 to 0.40) | -1.37 to 1.24 | RoB serious (unadjusted contrasts; source concerns); inconsistency serious; indirectness serious (heterogeneous SCH definitions; limited clinical applicability); imprecision serious (CI and/or PI span different directions). | Not assessable reliably (k<10). | Very low |
| Prolactin | 8/2180 | 0.52 (95% CI -0.27 to 1.32) | -1.81 to 2.85 | RoB serious (unadjusted contrasts; source concerns); inconsistency serious; indirectness serious (heterogeneous SCH definitions; limited clinical applicability); imprecision serious (CI and/or PI span different directions). | Not assessable reliably (k<10); influence instability. | Very low |
| Total testosterone | 9/2540 | 0.02 (95% CI -0.34 to 0.38) | -1.04 to 1.09 | RoB serious (unadjusted contrasts; source concerns); inconsistency serious; indirectness serious (heterogeneous SCH definitions; limited clinical applicability); imprecision serious (CI and/or PI span different directions). | Not assessable reliably (k<10). | Very low |
